# Higher expression of phosphorylated myosin regulatory light chain in the common bile duct in pancreaticobiliary maljunction accompanied by bile duct dilatation in children: a post-mortem observational study

**DOI:** 10.1007/s00383-012-3225-0

**Published:** 2012-12-09

**Authors:** Wan-liang Guo, Qi Zhang, Jian Wang, Mei-fang Jin

**Affiliations:** 1 Radiology Department, Children’s Hospital Affiliated to Soochow University, Suzhou, China; 2 Pediatric General Surgery Department, Children’s Hospital Affiliated to Soochow University, 215003 Suzhou, China

**Keywords:** Pancreaticobiliary maljunction (PBM), Common bile duct, Smooth muscle, Phosphorylated myosin regulatory light chain (P-MLC20), Myosin light-chain kinase (MLCK)

## Abstract

**Purpose:**

To examine the content of phosphorylated myosin regulatory light chain (P**-**MLC20) and myosin light-chain kinase (MLCK) in the common bile duct of pediatric patients with pancreaticobiliary maljunction (PBM) accompanied by bile duct dilatation (BDD), and investigate their potential role in PBM accompanied by BDD.

**Methods:**

Twenty-one specimens of the common bile duct from pediatric patients with PBM accompanied by BDD were collected. P-MLC20 was examined with immunohistochemistry. The expression of P-MLC20 and MLCK was also examined with Western blot. Twenty-one specimens of the common bile duct from pediatric patients without PBM and BDD were used as controls.

**Results:**

The mean optical density (MOD), mean labeling intensity (MLI) and minimum qualifying scores (MQS) of P-MLC20 were 115.6856 ± 58.1634, 21.7125 % ± 9.6555 and 21.3531 ± 6.5255, respectively. In the control group, MOD, MLI and MQS were 96.5581 ± 9.7859, 11.1813 % ± 3.6208 and 10.7819 ± 3.5323, respectively. There was no significant difference in MOD between the two groups (*P* > 0.05), whereas there was a significant difference in MLI and MQS between the two groups (*P* < 0.05). The expression of P-MLC20 and MLCK, as determined with Western blot, was also significantly higher in the PBM group than in the control group (*P* < 0.05).

**Conclusion:**

P-MLC20 is associated with increased contractile force of the smooth muscle of the common bile duct in pediatric patients with PBM accompanied by BDD. The enhanced expression of P**-**MLC20 in the common bile duct probably contributes to increased bile duct pressure in PBM via the MLCK pathway.

## Introduction

Pancreaticobiliary maljunction (PBM) is a congenital anomaly in which the junction of the pancreatic and bile ducts is outside the duodenal wall. Since the common duct of the pancreaticobiliary duct is too long, Oddi’s sphincter could not control the biliary–pancreatic confluence, and results in abnormal hydrodynamics in the biliary–pancreatic confluence [1, 2]. The pancreatic juice could reflux into the common bile duct due to high pressure within the pancreatic duct and lower pressure in the common bile duct. The bile activates a variety of enzymes, such as phospholipase A2 and proteases, in the pancreatic juice, and, leads to the breakdown, destruction and abscission of the epithelial elastic fiber of the common bile duct. Chronic stimulation of the common bile duct results in inflammation, thickening and fibrosis of the wall, epithelial hyperplasia, and even cancer of the common bile duct. The fibrosis of the common bile duct leads to poor bile flow, increased inner pressure in the common bile duct and cholestasis [3]. If pancreatic juice refluxes into the common bile duct, the bile flow is hampered, leading to spindle-shaped or cystic dilatation of the common bile duct. The morphological changes of the common bile duct alter the function and structure of the common bile duct wall [4]. However, the molecular mechanisms involved in smooth muscle contraction change of the common bile duct are still unclear.

Smooth muscle plays an important role in the common bile duct wall [5]. Smooth muscle contraction is induced by the sliding of thick against thin filaments. The intertwining of the two myosin heavy chains (MHC) forms the rod-shaped body and globular head of thick filaments [6]. The globular head possesses ATPase activity, and provides the energy for muscle contraction by ATP hydrolysis after binding to actin in the thin filaments. Myosin regulatory light chain (MLC20) is the primary regulatory domain of myosin. MLC20 functions by phosphorylating myosin light chain kinase (MLCK) and dephosphorylating myosin light chain phosphatase (MLCP) [7]. The main mechanism regulating smooth muscle contraction is the phosphorylation of MLC20, and the degree of phosphorylation of MLC20 determines the speed and intensity of muscle contraction [8]. Even mild change in the expression of P-MLC20 significantly influences smooth muscle contraction [9].

In the PBM patients, poor bile flow induces cholestasis. Increase of inner pressure of the common bile duct and the dilation of the common bile duct are the pathogenic basis of the common bile duct cysts. Watanabe et al. [10] reported that, in patients with congenital choledochal cyst (CCC) or PBM, a small number of the patients were found to show malignant transformation in their bile ducts in long-term follow-up, even if the CCC was removed. Todani et al. [11] reported that the residual extrahepatic bile duct continued to be at risk for malignant transformation after PBM surgery. The malignant transformation of the bile duct is primarily due to chronic bile duct inflammation induced by pancreatic juice reflux and destruction of the bile duct wall. Wang et al. [12] reported that cytokines in response to chronic inflammation could induce intestinal epithelial barrier dysfunction by increasing the phosphorylation of MLC. Most of PBM cases have chronic inflammation in bile duct wall [13]. Besides, the change of P-MLC20 can significantly influence smooth muscle contraction [9]. Thus, it is necessary to understand the expression and significance of P**-**MLC20 in pediatric patients with PBM accompanied by BDD.

## Materials and methods

### General information

This study was approved by the Ethics Committee of the Children’s Hospital affiliated to Soochow University. Written informed consent was obtained from legal guardians of the subjects following a detailed description of the purpose of the study. All experiments were carried out in strict accordance with the institution guidelines regarding the acquisition and experimental use of human tissues. Twenty-one specimens (dilatated common bile duct) from patients (10 males and 11 females) with PBM accompanied by BDD confirmed by imaging, surgery and pathology in the Children’s Hospital affiliated to Soochow University during a 3-year period from 2008 to 2011 were included in the current study. The age of patients ranged from 19 months to 10 years (median age:3 years). The diagnosis of PBM was defined as following [13, 14]: MRCP or IOC shows that the common duct (>5 mm) of the pancreaticobiliary duct converges outside the Oddi’s sphincter, with a biliary amylase concentration of >1,000 U/L. Twenty-one specimens of the common bile duct of newborn patients and pediatric patients who died due to diseases other than bile duct and pancreatic diseases (age range: 37 gestational weeks to 9 years, with a median gestational age of 2.8 years) were used as controls in immunohistochemistry test. The expression of P-MLC20 and MLCK of the common bile duct cystic wall of 12 patients with PBM accompanied by BDD and 9 patients (5 newborn and 4 pediatric patients) without PBM and BDD in the control group was also analyzed by Western blot.

### Methods

#### Immunohistochemistry

The expression of P-MLC20 was detected by an SP immunohistochemical method. Briefly, the tissue embedded in paraffin was serially cut at 4-μm sections. Sections were deparaffinized, hydrated to water and incubated with 3 % H_2_O_2_ for 30 min at room temperature to eliminate endogenous peroxidase. After rinsing with distilled water and immersion in PBS, antigen was retrieved using a microwave oven in citrate buffer (pH 6.0). Ten percent goat serum was used to block non-specific binding. The sections were incubated with the primary antibody (anti-p-MLC20 antibody, 1:200 dilution) overnight at 4 °C and for an additional 45 min at 37 °C. The sections were incubated with a biotinylated secondary antibody for 20 min at 37 °C and then horseradish peroxidase (HRP)-labeled streptavidin for 15 min before addition of 3,3′-diaminobenzidine (DAB). Stained slides were counterstained with hematoxylin and eosin (HE). The positive controls were supplied by LifeSpan Biosciences. In the positive controls, the smooth muscle of the common bile duct shows a spindle pattern, and the cytoplasm was stained yellow or brown (considered as positive). The primary antibody was omitted in the negative controls.

#### Analysis of immunohistochemistry images

Quantification of the immunohistochemical staining of P-MLC20 was carried out by two investigators blinded to the clinical conditions using a light microscope. Acquired images were analyzed by the IPP 6.0 image analysis software. For each slide, five random fields (400× magnification) were selected. Image analysis was carried out according to the methods described by Mady HH [15], and included the following: (1) labeling index (LI), i.e., the ratio of the positively stained area to the total area in interested regions. (2) Mean optical density (MOD), i.e., the ratio of the integral optical density to the total area of interested area. (3) A quick score (QS), calculated as the numeric product of the LI with MOD.

#### Western blot

For 100-mg tissue, 1 m of RIPA buffer and 10 ul of PMSF (phenylmethanesulfonylfluoride or phenylmethylsulfonyl fluoride) were used for homogenization. The homogenate was centrifuged at 12,000 rpm for 30 min and the supernatants were collected. The protein concentration was determined using a BCA method (Pierce, Rockford, IL). The supernatant was mixed with a 2× loading buffer and boiled for 5 min prior to SDS-PAGE (100 ug per lane). Separated proteins were transferred onto a NC membrane. After blocking the non-specific binding sites, samples were incubated with a monoclonal anti-P-MLC20 antibody (1:1,000 dilution, LifeSpan Biosciences) and a monoclonal anti-MLCK antibody (1:500 dilution, LifeSpan Biosciences) at 4 °C overnight, followed by incubation with a HRP-labeled secondary antibody. Protein bands were visualized with an ECL reagent. P-MLC20 and MLCK results were normalized against GAPDH.

### Statistical analysis

All data were processed by the SAS V8.0 software and are expressed as mean ± SD $( \overline{{{\upchi}}} \pm {\text{s}} )$. Based on the data distribution and variance homogeneity test, *t* test or Wilcoxon two-sample test was used to compare the data from the two groups. A *P* value <0.05 was considered statistically significant.

## Results

### The pathological changes in the common bile duct of pediatric patients with PBM accompanied by BDD

HE staining revealed infiltration of the dilated common bile duct with a large number of lymphocytes and mononuclear cells. No atypical hyperplasia was found in the PBM patients. In the PBM patients, the cystic wall of the common bile duct was composed of dense fibrous tissue, scattered elastic fibers and smooth muscle fibers. The epithelial lining of the cyst was damaged or even absent, with different amount of chronic inflammatory cell infiltration. Intestinal metaplasia was observed in some cases. The dilation common bile duct was cystic in 17 cases, spindle-shaped in two cases and cylindrical in the two remaining cases. The diameter of the common bile duct ranged from 1.2 to 6 cm, with a median diameter of 3 cm. The biliary starch enzyme concentration was 2,558–1,48,854 U/L (average 47,373 U/L).

### Immunohistochemistry

**Fig. 1:**
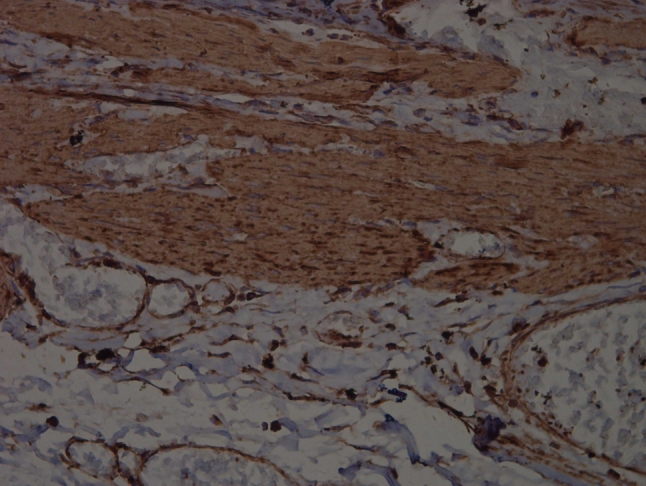
Representative immunohistochemical staining (×400) of P-MLC20 in a 5-year-old patient

**Fig. 2:**
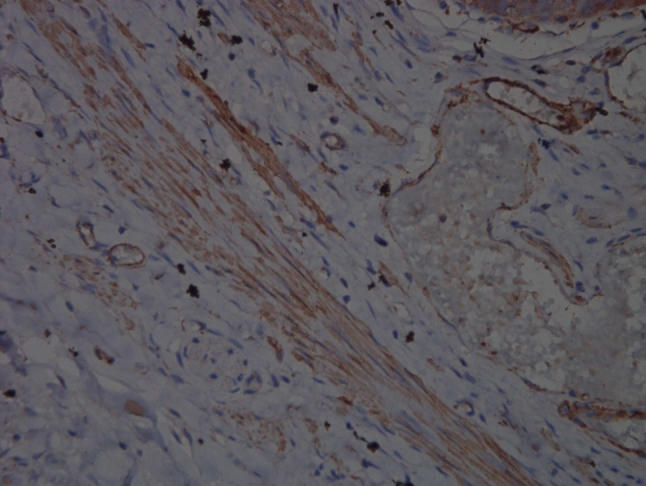
Representative immunohistochemical staining (×400) of P-MLC20 in a control subject

P-MLC20 was expressed mainly in the cytoplasm, with some positive nucleus (Figs. 1, 2). In the PBM group, the MOD, MLI and MQS of P-MLC20 were 115.6856 ± 58.1634, 21.7125 % ± 9.6555 and 21.3531 ± 6.5255, respectively. In the control group, the MOD, MLI and MQS of P-MLC20 were 96.5581 ± 9.7860, 11.1813 % ± 3.6208 and 10.7819 ± 3.5323, respectively. There was no significant difference in MOD between the two groups (*P* > 0.05), whereas there was a significant difference of MLI and MQS between the two groups (*P* < 0.05) (Table 1).

**Table 1:** Image analysis of P-MLC20 expression in common bile duct in different groups

Image analysis data	PBM group (*n* = 21)	Control group (*n* = 21)	*Z*	*P* value
MOD	115.6856 ± 58.1634	96.5581 ± 9.7859	−0.7726	0.4456
MLI (%)	21.7125 ± 9.6554	11.1813 ± 3.6208	3.5239	0.0004
MQS	21.3531 ± 6.5255	10.7819 ± 3.5323	4.3154	0.0002

Wilcoxon two-sample test statistically significant (*P* < 0.05)

#### P-MLC20 expression by Western blot

**Fig. 3:**
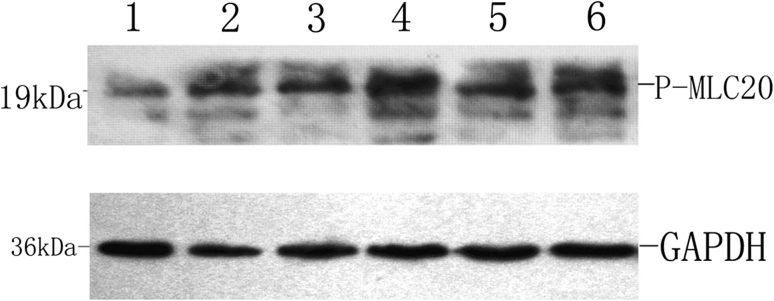
Representative Western blot of P-MLC20 in the PBM group (*4*, *5*, *6*) versus the control (*1*, *2*, *3*)

The relative expression of P-MLC20 was significantly higher in the PBM group (0.6791 ± 0.3121) than in the control group (0.3433 ± 0.2943; *P* < 0.05) (Table 2, Fig. 3).

**Table 2:** Analysis of P-MLC20 expression by Western blot in pancreaticobiliary maljunction accompanied by bile duct dilatation in children versus control

Groups	Cases	$\overline{{{\upchi}}} \pm {\text{s}}$	*Z*	*P*
PBM + BDD	12	0.6791 ± 0.3121	−2.3097	0.0209
Control	9	0.3433 ± 0.2943		

Wilcoxon two-sample test

### MLCK expression by Western blot

**Fig. 4:**
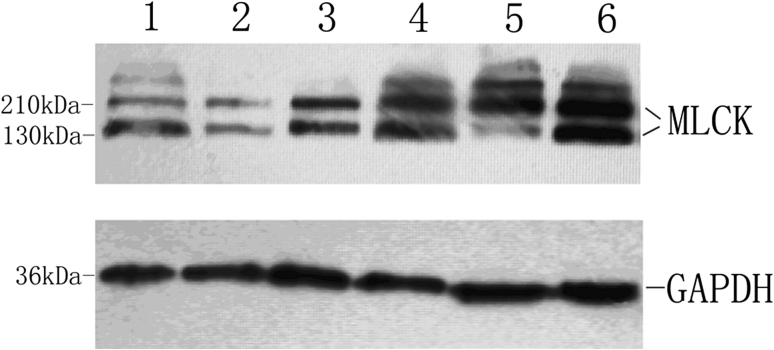
Representative Western blot of MLCK in the PBM group (*4*, *5*, *6*) versus the control group (*1*, *2*, *3*)

The expression of MLCK was significantly higher in the PBM group (1.8993 ± 1.1931) than in the control group (1.0695 ± 0.8977) (*P* < 0.05) (Table 3, Fig. 4).

**Table 3:** Analysis of MLCK expression by Western blot in pancreaticobiliary maljunction accompanied by bile duct dilatation in children versus control

Groups	Cases	$\overline{{{\upchi}}} \pm {\text{s}}$	*Z*	*P*
PBM + BDD	12	1.8993 ± 1.1931	−2.2386	0.0252
Control	9	1.0695 ± 0.8977		

Wilcoxon two-sample test

## Discussion

Poor bile flow in PBM induces cholestasis, and results deconstruction of the bile duct wall, as well as changes in the bile ducts inside and outside the liver. Cholestasis induced by fibrosis of the common bile duct could increase the pressure in the common bile duct and the dilation of the common bile duct, and eventually common bile duct cysts. Alonso-Lej et al. [4] reported that the common bile duct wall is mainly composed of compact fibrous tissue, smooth muscle and elastic fibers, and the bile duct wall in patients with congenital bile duct dilation is thickened up to a diameter of about 1 cm, suggesting that the changes in morphology and function of the common bile duct is accompanied by the changes in the tissue structure.

Myosin is a part of the skeleton structure of common bile duct and pancreatic duct. The myosin–actin interaction takes part in many biological processes, including muscle contraction, cell motility, membrane transport of materials and specific cell signal transduction [16]. Smooth muscle contraction is induced by the sliding of thick against the thin filaments. Similar to skeletal muscle, the myosin in smooth muscle cell is composed of two heavy chains and four light chains (two MLC20 and two LC17) [7]. The intertwining of the two MHC forms the rod-shaped body and globular head of thick filaments [6]. The globular head has ATPase activity, and provides the energy for the relative sliding of thick and thin filaments by ATP hydrolysis. The above complex is dependent on myosin-Ca^2+^-ATPase. The myosin light chain is subjected to regulation via phosphorylation [17]. The myosin light chain is composed of two regulatory light chains and basic light chains. The phosphorylation of MLC20 is the main pathway that promotes smooth muscle contraction [8].

The phosphorylation of MLC20 depends on the relative activity of MLCK and MLCP. The regulatory pathways of MLCP/MLCK include a calcium-dependent pathway and a calcium-independent pathway [7]. It is generally accepted that MLCK is activated after Ca^2+^ binding to calmodulin, and MLCK is most readily phosphorylated at Serine 19 of MLC20 [18]. The second most commonly phosphorylation site is Serine 18. The phosphorylation activates the Ca^2+^-ATPase, which hydrolyzes ATP providing energy for the interaction of myosin and actin, leading to muscle contraction. The region at which MLCK exerts its action is located at the central kinase region, and the phosphorylation of MLC20 and Ca^2+^ are required for inducing muscle contraction [19].

Our results indicated a significant difference of MLI and MQS between the two groups (*P* < 0.05), but not MOD. Furukawa et al. [20] suggested that it is more reasonable to take both the positively stained area and stained intensity into account for image analysis to limit the influence of inter-individual variability. In the current study, we used a MQS method developed by Hussan H et al. [15]. The results showed that the P-MLC20 expression was increased in the common bile duct in patients with PBM accompanied by BDD. Higher expression of P-MLC20 was confirmed by Western blot analysis. Pfitzer and Arner [9] reported that the content of phosphorylated MLC20 closely correlated to muscle contractile force. If there was a change of 8 % in P-MLC20, the change of muscle contraction could be as high as 80 %. We speculate that the contractile force of the smooth muscle of the common bile duct in PBM patients is enhanced, which in turn triggers or aggravates bile reflux to pancreatic duct in PBM. Most of PBM cases have chronic inflammation in bile duct wall [13]. Wang et al. [12] reported that cytokine release in response to chronic inflammation induces intestinal epithelial barrier dysfunction via increased phosphorylation of MLC. High expression of P-MLC20 in common bile duct in PBM accompanied by BDD could be secondary to the chronic inflammation, and exacerbates the inflammation, thus forming a vicious cycle. The likelihood that high expression of P-MLC20 causes PBM, however, cannot be ruled out.

The content of myosin regulatory light chains decreases in patients with end-stage expansionary cardiomyopathy [21]. The reduction of myosin regulatory light chains could decrease the dissociation speed and affect muscle contraction [22, 23]. MLCK is expressed in a variety of non-muscle eukaryotic cells. MLCK is composed of four regions: N-terminal actin-binding domain, central kinase domain, calmodulin-binding domain and C-terminal myosin-binding domain [24]. In smooth muscle contraction, phosphorylation of MLC20 is regulated by MLCK pathway, ZIP kinase pathway and integrin-related kinase pathway [25, 26]. In our study, the MLCK expression was significantly higher in PBM accompanied by BDD, suggesting the contraction of the smooth muscle of the common bile duct is dependent on the phosphorylation of MLC20 regulated by MLCK pathway. With enhanced expression of phosphorylated MLC20, the contractile force of the smooth muscle of the common bile duct is enhanced. Using cultured gastric antral cells and smooth muscle cells of the proximal small intestine, Huang et al. [27] evaluated the role of G protein-dependent signaling pathway in regulating the initiation and maintenance of smooth muscle contraction and phosphorylation of MLC20. The results indicated that motilin initiates a transient Gαq-mediated signaling cascade involving IP_3_-dependent Ca^2+^ release and MLCK-dependent phosphorylation of MLC20. Motilin acts on Gαq and Gα13-mediated signaling cascade involving Rho kinase and PKC-dependent pathway, and results in persistent phosphorylation of MLC20 and smooth muscle contraction.

Improving the bile flow from the bile duct at resting state is of vital importance for reducing the damages of the bile duct, liver and pancreas. P-MLC20 expression was enhanced in PBM accompanied by BDD patients. However, changes in the expression of MLC basic light chains remains unclear.

In summary, we found that, with the occurrence of biliary pancreatic reflux in pediatric patients with PBM accompanied by BDD, the morphology of the common bile duct was altered. P-MLC20 expression was increased, and possibly contributed to increased contractile force of the smooth muscle of the common bile duct by phosphorylating MLC20 via the MLCK pathway.

## References

[1] Kamisawa T, Tu Y, Nakajima H, Egawa N, Tsuruta K, Okamoto A (2007). The presence of a common channel and associated pancreaticobiliary diseases: a prospective ERCP study. Dig Liver Dis.

[2] Kamisawa T, Egawa N, Nakajima H, Tsuruta K, Okamoto A, Matsukawa M (2005). Origin of the long common channel based on pancreatographic findings in pancreaticobiliary maljunction. Dig Liver Dis.

[3] Watanabe Y, Kubota H, Honma T, Hosoya T, Yamaguchi K (1997). Usefulness of helical DIC-CT in pancreaticobiliary maljunction. Nippon Igaku Hoshasen Gakkai Zasshi (Japanese).

[4] Alonso-Lej Fernando, Rever WB (1959). Congenital choledochal cyst with a report of two and an analysis of ninety-four cases. Int Abstr Surg.

[5] Ludwick JR (1967). Observations on the smooth muscle and contractile activity of the common bile duct. Ann Surg.

[6] Rayment I, Holden HM, Whittaker M, Yohn CB, Lorenz M, Holmes KC, Milligan RA (1993). Structure of the actin–myosin complex and its implications for muscle contraction. Science.

[7] Somlyo AP, Somlyo AV (1994). Signal transduction and regulation in smooth muscle. Nature.

[8] Hai C-M, Murphy RA (1989). Ca^2+^, cross-bridge phosphorylation, and contraction. Annu Rev Physiol.

[9] Pfitzer G, Arner A (1998). Involvement of small GTPases in the regulation of smooth muscle contraction. Acta Physiol Scand.

[10] Watanabe Y, Toki A, Todani T (1999). Bile duct cancer developed after cyst excision for choledochal cyst. J Hepatobiliary Pancreat Surg.

[11] Todani T, Watanabe Y, Urushihara N (1994). Choledochal cyst, pancreatobiliary malunion, and cancer. J Hep Bil Pancr Surg.

[12] Wang F, Graham WV, Wang Y, Witkowski ED, Schwarz BT, Turner JR (2005). Interferon-gamma and tumor necrosis factor-alpha synergize to induce intestinal epithelial barrier dysfunction by up-regulating myosin light chain kinase expression. Am J Pathol.

[13] Guo WL, Huang SG, Wang J, Sheng M, Fang L (2012). Imaging findings in 75 pediatric patients with pancreaticobiliary maljunction: a retrospective case study. Pediatr Surg Int.

[14] Misra SP, Gulati P, Thorat VK, Vij JC, Anand BS (1989). Pancreaticobiliary ductal union in biliary diseases. An endoscopic retrograde cholangiopancreaticographic study. Gastroenterology.

[15] Mady HH, Melhem MF (2002). FHIT protein expression and its relation to apoptosis, tumor histologic grade and prognosis in colorectal adenocarcinoma: an immunohistochemical and image analysis study. Clin Exp Metastasis.

[16] Vale RD, Milligan RA (2000). The way things move: looking under the hood of molecular motor proteins. Science.

[17] Fugimoto K, Yasue H, Nakao K, Yamamoto H, Hitoshi Y, Jougasaki M, Okumura K, Ogawa H, Takatsu K, Miyamoto E (1993). Novel monoclonal antibodies specific for human cardiac myosin light chain-1: useful tools for analysis of normal and pathological hearts. J Histochem Cytochem.

[18] Hirano K, Derkach DN, Hirano M, Nishimura J, Kanaide H (2003). Protein kinase network in the regulation of phosphorylation and dephosphorylation of smooth muscle myosin light chain. Mol Cell Biochem.

[19] Murahashi T, Fujita A, Kitazawa T (1999). Ca^2+^ -induced Ca^2+^ desensitization of myosin light chain phosphorylation and contraction in phasic smooth muscle. Mol Cell Biochem.

[20] Furukawa Y, Kimijima I, Abe R (1998). Immunohistochemical image analysis of estrogen and progesterone receptors in breast cancer. Breast Cancer.

[21] Schaper J, Froede R, Hein S, Buck A, Hashizume H, Speiser B, Friedl A, Bleese N (1991). Impairment of the myocardial ultrastructure and changes in the cytoskeleton in dilated cardiomyopathy. Circulation.

[22] Solaro RJ (1992). Myosin and why hearts fail. Circulation.

[23] Hofmann PA, Metzger JM, Greaser ML, Moss RL (1990). The effect of partial extraction of light chain 2 on the Ca^2+^ sensitivities of isometric tension stiffness and velocity of shortening in skinned skeletal muscle fibers. J Gen Physiol.

[24] Nakamura A, Xie C, Zhang Y, Gao Y, Wang HH, Ye LH, Kishi H, Okagaki T, Yoshiyama S, Hayakawa K, Ishikawa R, Kohama K (2008). Role of non-kinase activity of myosin light-chain kinase in regulating smooth muscle contraction, a review dedicated to Dr. Setsuro Ebashi. Biochem Biophys Res Commun.

[25] Haystead TA (2005). ZIP kinase, a key regulator of myosin protein phosphatase 1. Cell Signal.

[26] Ho B, Bendeck MP (2009). Integrin linked kinase (ILK) expression and function in vascular smooth muscle cells. Cell Adh Migr.

[27] Huang J, Zhou H, Mahavadi S, Sriwai W, Lyall V, Murthy KS (2005). Signaling pathways mediating gastrointestinal smooth muscle contraction and MLC20 phosphorylation by motilin receptors. Am J Physiol Gastrointest Liver Physiol.

